# Supplementary material for: Identification of photoperiod-induced specific miRNAs in the adrenal glands of Sunite sheep (Ovis aries)
Source: Front Vet Sci. 2022 Jul 22;9:888207. doi: 10.3389/fvets.2022.888207 (PMC9354845; doi:10.3389/fvets.2022.888207)
Supplement: Supplementary file 1 [file Data_Sheet_1.ZIP › Supplementary file/miRDeep2 file.zip!/miRDeep2 file/Know_Structure/LP42A1/oar-mir-26a.pdf]

miRBase precursor : oar-mir-26a
Total read count : 1398228
oar-miR-26a read count : 1398071
remaining reads : 157

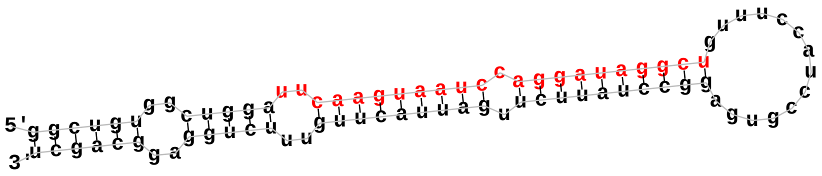

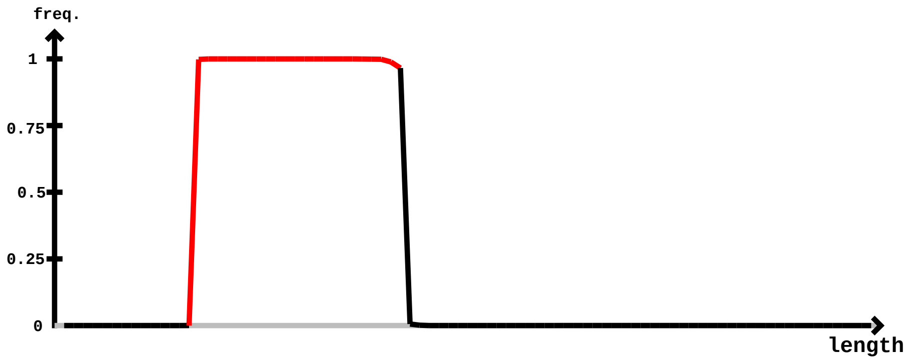


```
                oar-miR-26a
5'- ggcuguggcuggauucaaguaauccaggauaggcuguuuccauccgugaggccuauucuugauuacuuguuucuggaggcagcu  -3'    exp
    ((((((..(((((..(((((((((.((((((((((..............))))))))))).)))))))))..)))))..))))))   reads      mm        sample
    ............auucaaguaauccaggauagg...................................................   3          0         seq
    ............auucaaguaauccaggauaggc..................................................   15         0         seq
    ............Guucaaguaauccaggauaggc..................................................   1          1         seq
    ............auucaaguaauccaggauaggcu.................................................   224        0         seq
    ............Guucaaguaauccaggauaggcu.................................................   44         1         seq
    .............uucaaguaauccaggaG......................................................   14         1         seq
    .............uucaagGaauccaggau......................................................   2          1         seq
    .............uucaaguaauccaggaA......................................................   19         1         seq
    .............uucaaguaauccaggau......................................................   476        0         seq
    .............uucaGguaauccaggau......................................................   3          1         seq
    .............uAcaaguaauccaggau......................................................   1          1         seq
    .............uucaUguaauccaggau......................................................   2          1         seq
    .............uucaaguaUuccaggau......................................................   1          1         seq
    .............uucaaguaauccaggCu......................................................   1          1         seq
    .............uucaaAuaauccaggaua.....................................................   2          1         seq
    .............uucaaguaauccaggaua.....................................................   657        0         seq
    .............uucaGguaauccaggaua.....................................................   1          1         seq
    .............uucaaguaauccaggauaC....................................................   7          1         seq
    .............uucaaguaauccaggauag....................................................   756        0         seq
    .............uucaaguaauccaggauagg...................................................   13764      0         seq
    .............uucaaguaauccaggauaggc..................................................   32161      0         seq
    .............uucaaguaauccaggauaggcu.................................................   1339309    0         seq
    .............uucaaguaauccaggauaggcug................................................   6033       0         seq
    .............uucaaguaauccaggauaggcugu...............................................   1952       0         seq
    .............uucaaguaauccaggauaggcuguG..............................................   7          1         seq
    .............uucaaguaauccaggauaggcuguu..............................................   28         0         seq
    .............uucaaguaauccaggauaggcuguuu.............................................   2          0         seq
    ..............ucaaguaauccaggauag....................................................   3          0         seq
    ..............ucaaguaauccaggauagg...................................................   16         0         seq
    ..............ucaaguaauccaggauaggc..................................................   59         0         seq
    ..............ucaaguaauccaggauaggcu.................................................   2353       0         seq
    ..............ucaaguaauccaggauaggcug................................................   22         0         seq
    ..............ucaaguaauccaggauaggcugu...............................................   5          0         seq
    ...............caaguaauccaggauagg...................................................   1          0         seq
    ...............caaguaauccaggauaggc..................................................   4          0         seq
```

oar-miR-26a

```
ggcuguggcuggauucaaguaauccaggauaggcuguuuccauccgugaggccuauucuugauuacuuguuucuggaggcagcu
...............caaguaauccaggauaggcu.................................................   105   0   seq
...............caaguaauccaggauaggcug................................................   2     0   seq
................aaguaauccaggauaggc..................................................   1     0   seq
................aaguaauccaggauaggcu.................................................   14    0   seq
.................aguaauccaggauaggcu.................................................   1     0   seq
................................................aggccuauucuugauuacuugu..............   3     0   seq
................................................aggccuauucuugauuacuuguu.............   2     0   seq
................................................aggccuauucuugauuacuuguuu............   1     0   seq
...................................................ccuauucuugauuacuugu..............   1     0   seq
...................................................ccuauucuugauuacuuguu.............   5     0   seq
...................................................ccuauucuugauuacuuguuu............   73    0   seq
...................................................ccuauucuugauuacuuguuuc...........   65    0   seq
...................................................ccuauucuugauuacuuguuucu..........   2     0   seq
....................................................cuauucuugauuacuuguuu............   3     0   seq
....................................................cuauucuugauuacuuguuuc...........   1     0   seq
....................................................cuauucuugauuacuuguuucu..........   1     0   seq
```
